# Synthesis of a Au/Au NPs-PPy/l-CYs/ZIF-8 nanocomposite electrode for voltammetric determination of insulin in human blood

**DOI:** 10.1039/d3ra04064j

**Published:** 2023-08-16

**Authors:** Jamal Kouhdareh, Rahman Karimi-Nami, Hassan Keypour, Khadijeh Rabiei, Sedigheh Alavinia, Shokoufeh Ghahri Saremi, Mohammad Noroozi

**Affiliations:** a Faculty of Chemistry, Bu-Ali Sina University Hamedan 65174 Iran haskey1@yahoo.com; b Department of Chemistry, Faculty of Science, University of Maragheh Maragheh Iran; c Department of Chemistry, Faculty of Science, Qom University of Technology Qom Iran; d Department of Chemistry, Payame Noor University Tehran Iran; e Center for Research and Development of Petroleum Technologies at Kermanshah, Research Institute of Petroleum Industry (RIPI) Iran

## Abstract

In this work, a modified electrode named Au/Au NPs-PPy/l-CYs/ZIF-8 was designed and built and simultaneously doped into electropolymerized polypyrrole (PPy) film using cyclic voltammetry (CV). Scanning Electron Microscopy (SEM), Electrochemical Impedance Spectroscopy (EIS), and CV were used to characterize the composite films. The PPy-(ZIF-8) modified Au electrode was used to determine insulin using Square-Wave Voltammetry (SWV). It was found that the prepared zeolitic imidazolate framework-8 had excellent electrocatalytic activity towards insulin oxidation due to its unique properties. The oxidation peak current of insulin hormone increased with its concentration in the range from 1.0 to 60 nM with the linear regression equation: *I*_pa_ = 0.3421*C* (nM) + 3.2762 (γ = 0.998). The measurement limit was estimated to be 1 nM. While the common coexisting substances showed no interference in the response of the modified electrode to insulin, the modified electrode indicated reproducible behavior and a high level of stability during the experiments. The advantages of using these nanocomposites on the surface of modified electrodes include increased stability, good interaction between the analyte and the modified electrode, conductivity, and excellent performance due to the nanometer size of the composites. As a result, it may be particularly suitable for analytical purposes.

## Abstract 2

In this work, a modified electrode named Au/Au NPs-PPy/l-CYs/ZIF-8 was designed and built and simultaneously doped into electropolymerized polypyrrole (PPy) film using cyclic voltammetry (CV).

## Graphical abstract

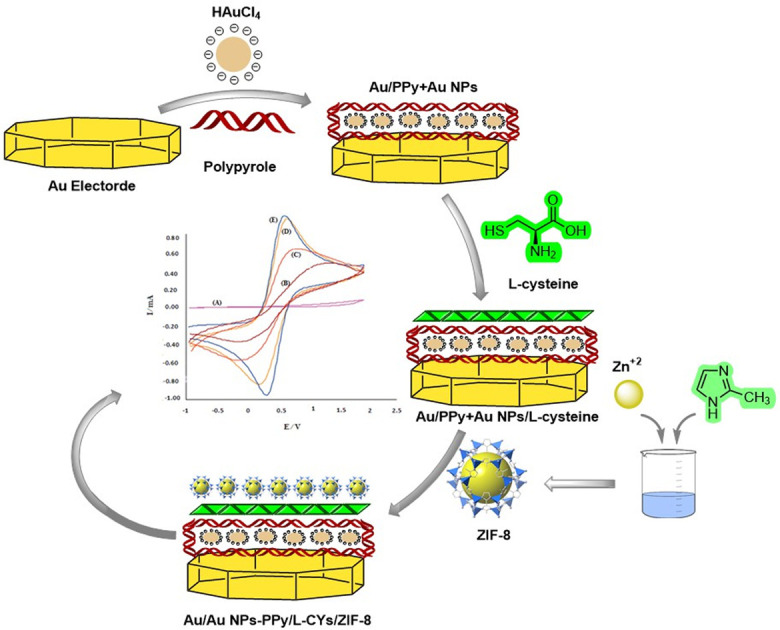

## 1 Introduction

Insulin is a hormone made in the pancreas, a gland located behind the stomach, and is an essential polypeptide hormone that controls glucose levels in the blood. It is used for the treatment of insulin-dependent type I diabetes. The standard analytical methods for insulin identification and measurement in serum samples include bioassays, immunoassays, and chromatography. Direct and rapid electrochemical measurement of insulin is therefore essential and crucial. Some previous studies have already been reported about *in vitro* electrochemical insulin measurement using modified common electrodes as a sensing platform, which is a cheaper method with higher efficiency and lower cost.^1–3^

However, direct electrochemical measurement of insulin is fast, simple, and of low-cost. Also, it is attractive as it can provide sensitivity and reduces analysis time to enable frequent real-time measurements compared to the standard analytical methods. Biosensor-related research and studies have experienced explosive growth over less than two decades. An electrochemical biosensor is an analytical sensing device that transduces biochemical events to electrical signals.^4,5^ The purpose of designing and manufacturing a chemical sensor is to provide reliable information about the chemical composition and structure in the shortest possible time. Ideally, such a device can respond continuously and reversibly and does not perturb the sample. This class of sensors consists of a transmission element coated with a chemical and biological detection layer or film composite. In the case of electrochemical sensors, the analytical information is obtained from the electrical signal that results from the interaction of the target analyte and the recognition layer. To investigate the electrochemical oxidation of insulin hormone, various chemically modified electrodes have been made with features such as being fast response, inexpensive, and measurement *in situ* conditions.^6–9^ Zeolitic imidazolate frameworks (ZIFs) and Metal–organic frameworks (MOFs) have received significant attention in the last decade due to their unique properties different from those of bulk materials, such as top conductivities, outstanding electrocatalytic activities, optical and electrochemical properties.^10–19^ Notably, gold nanoparticles (GNPs) have been widely used in many electrochemical fields due to their potential for catalysis, mass transport, and high effective surface area.^20^ The catalytic performance of GNPs significantly depends on the structure of the local microenvironment.^21–23^ ZIF-8 based on Zn^2+^ has been extensively studied in the drug delivery system due to its low toxicity and good biocompatibility.^24^

Zeolitic imidazolate frameworks (ZIFs) show conductivity properties according to excellent electrical conductivity above their inherent qualities, highly available specific surface, and metal coordination to organic ligands.^25–27^ Transition metals are essential in biological systems because they have multiple oxidation states separated by only modest potentials, which make them suitable candidates for electron-transfer processes. ZIF-8 shows high catalytic activity in the oxidation of some organic compounds compared to other (incredibly noble metal) catalysts inexpensively.^28–30^ Keypour *et al.* reported a new Schiff base functionalized magnetic Fe_3_O_4_ nanoparticle/MWCNTs modified glassy carbon electrode to determine citalopram in human blood serum samples.^4^

In this work, we have reported the synthesis of a PPy-(ZIF-8) modified Au electrode prepared by the modifier's electrochemical-pulsed potential deposition and the application of its electrochemical behavior in the voltammetric determination of insulin. The zeolitic imidazolate framework-8 (ZIF-8) was synthesized based on previous reports, and it was proposed to be fully compatible with the criteria of making an excellent sensor.^31^

## 2 Experimental

### 2.1 Reagents and chemicals

All compounds and chemicals used in this project were obtained from Merck and Sigma companies. Britton–Robinson buffer (aka BRB aka PEM) is a “universal” pH buffer used for the pH range from 2.0 to 12.0. Data from electrochemical interactions were performed using an auto-lab potentiostat model PGstat302N (Metrohm). The working system consists of three electrodes comprised of a modified working electrode as Au/Au-PPy-ZIF-8, a saturated Ag/AgCl electrode as a reference electrode, and a platinum wire as a counter electrode. All reported potentials are based on Ag/AgCl reference criteria. A scanning electron microscope (SEM, PhilipsXL30) with gold coating characterized the surface morphology of modified electrodes.

### 2.2 Synthesis of zeolitic imidazolate framework-8 (ZIF-8)

**Fig. 1:**
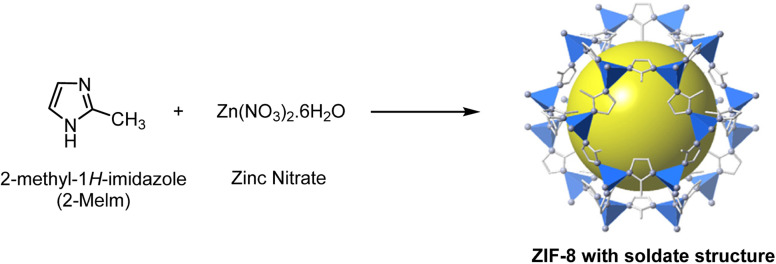
Crystal structure of ZIF-8: Zn (polyhedral), N (sphere), and C (line).

In Fig. 1, the schematic route of ZIF-8 synthesis was provided by following the Pan Y., *et al.* and colleagues' method in an aqueous solution at room temperature.^32^ In the initial step, the solution was prepared with a molar ratio of Zn^2+^ : 2-methyl-2*H*-imidazole : water = 1 : 70 : 1238. Next, we dissolved 0.585 g of zinc nitrate in 4 mL of distilled water. In the next step, 11.35 g of 2-methyl-2*H*-imidazole was dissolved in 40 mL of distilled water, and then 6 mL of DMSO was added to it. Then, zinc nitrate solution was added to the ligand solution, and a milky white solution was formed quickly. The synthesized solution was centrifuged, and after sedimentation, we washed the sediment several times with distilled water to remove unreacted and excess materials and dried it in the oven at 60 °C for 24 h.^33–36^

### 2.3 Electrode modification

**Fig. 2:**
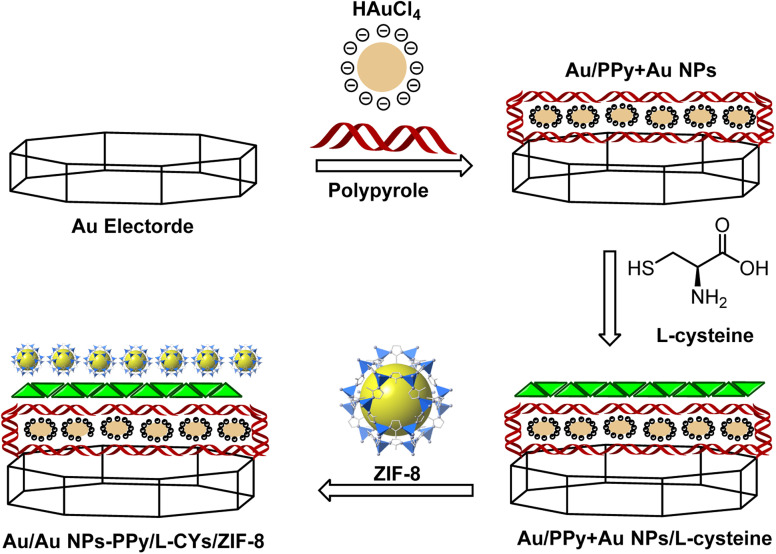
Overall scheme for the preparation of Au/Au NPs-PPy/l-CYs/ZIF-8 nanocomposite electrode.

The general schematic of modification of the Au bare electrode is shown in Fig. 2. For electrode modification, the emery paper was used for polishing the Au bare electrode, followed by alumina (1.0 and 0.05 mm), and deionized water was applied for washing thoroughly. Then, the Au nude electrode was placed in a beaker containing water and ethanol and transferred to an ultrasonic bath to remove contamination on the electrode surface. The Au bare electrode was immersed into the solution containing 2 mL of 3% (v/v) PPy and 2 mL of 1 mM of HAuCl_4_·3H_2_O in 25 mL of deionized water under applying cyclic voltammograms from +1.5 to −1.5 V (*N* = 12). The modified Au/Au NPs-PPy was preserved in a solution of l-cysteine (5 mol L^−1^) for 24 h at room temperature to produce Au/Au NPs-PPy/l-CYs. The modified electrode was immersed in deionized water to repulse physically absorbed l-CYs. In a separate experiment, 35 mg of trifluoromethane sulfonic anhydride (TF_2_O) (0.125 mmol) and 0.05 g of ZIF-8 were mixed well by the ultrasonic bath at 40 °C. Afterward, for 6 h, the modified Au/Au NPs-PPy/l-CYs electrode was dipped into the dispersed solution containing ZIF-8 under stirring. Therefore, the final Au/Au NPs-PPy/l-CYs/ZIF-8 was prepared and used to determine insulin hormone.

### 2.4 Human serum sample preparation

Serum samples were obtained from a healthy volunteer and stored frozen until assay. After gently thawing, an aliquot sample volume was fortified with RBV dissolved in bidistilled water to achieve appropriate concentration. The solution was centrifuged for 30 min at 3600 rpm to remove the precipitated serum protein, and the supernatant was taken carefully. The appropriate volume of the supernatant liquor was transferred to the voltammetric cell.^37^

### 2.5 The vial hormone preparation

The proposed built electrode analyzed hormone vial formulations containing insulin. 5 vials containing 1.30 mg (4 IU) per one of insulin were mixed well to calculate the average vial contents. One-fifth of the solution was taken, transferred to a 100 mL bottle, and made up to volume with 0.5 mol L^−1^ NaNO_3._ The using Briton–Robinson buffer in the pH range was stabilized at 7.0. 1 mL of this solution was transferred to the volumetric flask and was made up to 100 mL with distilled water. This diluted solution was used in quantitative and qualitative analysis.

## 3 Results and discussion

### 3.1 Characterization of the Au/Au NPs-PPy/l-CYs/ZIF-8 nanocomposite electrode

**Fig. 3:**
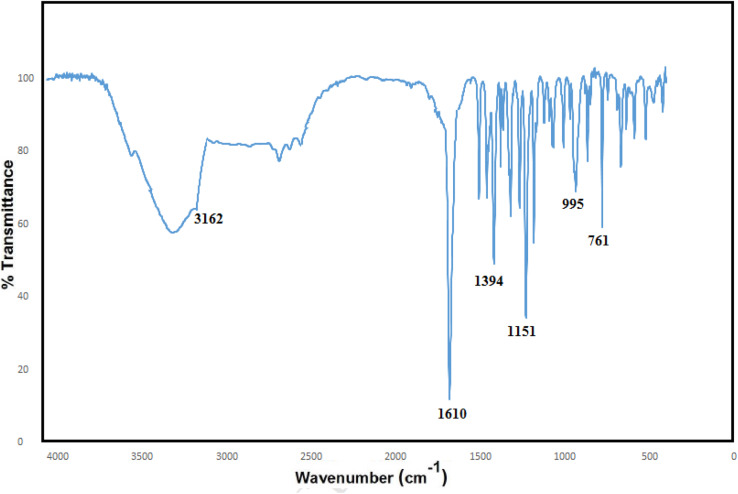
FT-IR spectra of ZIF-8.

In the FT-IR spectrum of the ZIF-8, a broad peak in the region of 3500 cm^−1^ is related to the stretching bond of N–H, and the small peak in the area of 3162 cm^−1^ can be attributed to C–H stretching in the vibrational modes inside the imidazole ring which is attached to the methyl group.^38–40^ The long peak in the region of 1610 cm^−1^ can be related to the C=N stretching states, and the peak in the area of 1394 cm^−1^ is associated with the stretching inside the ring.^41^ The peak is observed in the region at 480 cm^−1^, which is related to the Zn–N stretching in the ZIF-8 structure because the zinc atoms in the ZIF-8 structure bond to the nitrogen atoms in 2-methyl-2*H*-imidazole; they are connected in the direction of ZIF-8 formation (Fig. 3).

**Fig. 4:**
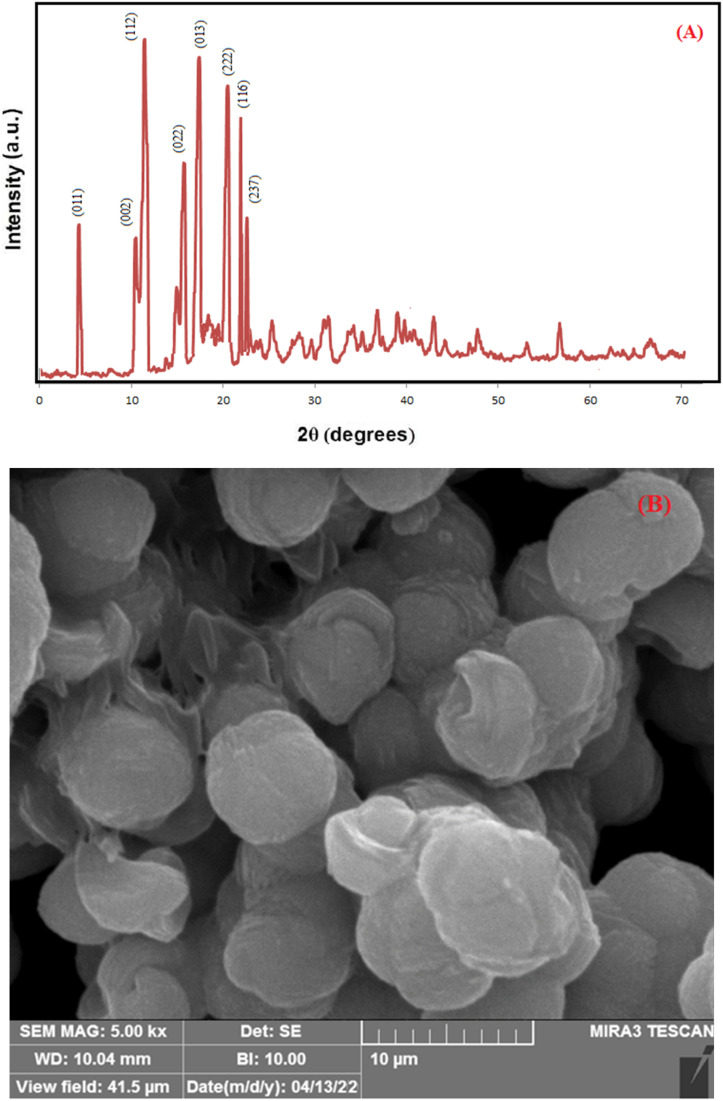
XRD patterns for simulated ZIF-8 (JCPDS 00-062-1030) (A), FESEM micrographs of (B) pure ZIF-8.

The overall XRD pattern of ZIF-8 (A) obtained with characteristic peaks (011), (002), (112), (022), (013), (222), (116), and (237) was in good agreement with the literature values and confirmed the formation of ZIF-8 crystal structure^42–44^ (Fig. 4A). Several diffraction peaks confirm the polycrystalline step in the pattern. From the XRD pattern, (*C*_s_) is the average crystallite size, and the dislocation density is (*D*) can be determined as follows: eqn (1) and (2).1
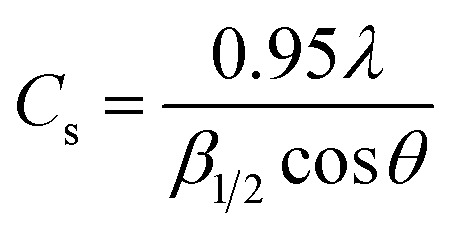
2
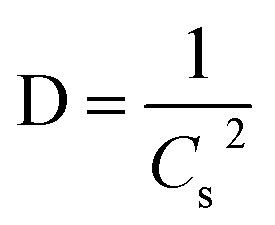
\where *λ* is the wavelength of X-ray radiation (*λ* = 1.5406 Å), *θ* is the angle of diffraction, and *β*_1/2_ is the complete width measured in radians at half the maximum reference diffraction peak.^45,46^ The obtained value is 100 nm, consistent with the result measured from the SEM images.^12,47^ In Fig. 4B, the morphology of the ZIF-8 surface can be seen by scanning electron microscope (SEM), which is consistent with the previously synthesized samples.^48^

**Fig. 5:**
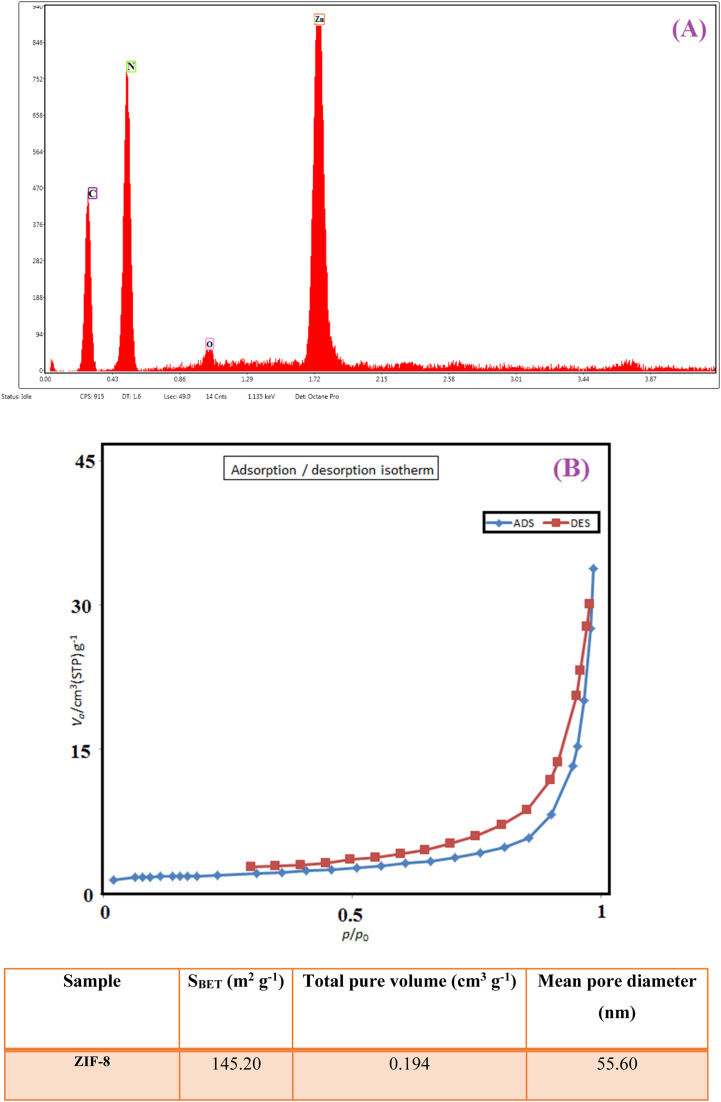
Energy dispersive X-ray analysis (EDX) ZIF-8 (A) Brunauer–Emmett–Teller (BET) (B) ZIF-8.

In Fig. 5A, the result of the energy dispersive X-ray analysis (EDX) of ZIF-8 indicates the presence of expected elements of C, N, and Zn in the crystal structure of the ZIF-8 composition. The presence of a small amount of oxygen atoms can be due to the presence of some water molecules encapsulated in ZIF-8 cavities. N_2_ adsorption/desorption techniques were used to determine the surface structural parameters. Fig. 5B shows the result of the N_2_ adsorption/desorption analysis related to the composition of ZIF-8. The surface area obtained based on the BET isotherm is 145.20 m^2^ g^−1^, and the total pore volume of the catalyst is 0.194 cm^3^ g^−1^. The adsorption isotherm is of type III, and the appearance of a hysteresis loop indicates the presence of mesopores in the sample.^49^

### 3.2 Electrochemical characterization of the Au/Au NPs-PPy/l-CYs/ZIF-8 nanocomposite film

In biosensors, information is extracted by measuring electrical properties to determine the electrochemical nature of the target element. This transfer of information and identification is due to the biocatalytic activity of biological compounds with the desired sensor.^50,51^ The electrochemical behavior of Au-PPy − Au/PPy + Au NPs/l-cysteine/ZIF-8 crystalline structure toward insulin was studied using square-wave (SW) and cyclic voltammetry (CV). CV measurements, insulin gave a well-defined cathodic peak in the serum sample, indicating that bonds N–Zn were reduced. Electropolymerization is widely accepted as an appropriate methodology for preparing suitable nanomaterials. Electropolymerization is one of the proper methods for preparing nanomaterials or loading them on surfaces. The main advantage of this method is that the thickness or diameter of the layers of the synthesized particles can be controlled. The increase of ZIF-8 in this polymer matrix increases the electrochemical activity/conductivity due to the high available cross-sectional area and excellent conductivity in this substrate. So, cyclic voltammograms of analyzes of 20 nM of insulin response at a scanning rate of 100 mV s^−1^ on the surface of different film sensors such as Au-PPy – Au/PPy + Au NPs/l-cysteine/ZIF-8 crystalline structure electrodes were performed in pH 7.00.^52^

**Fig. 6:**
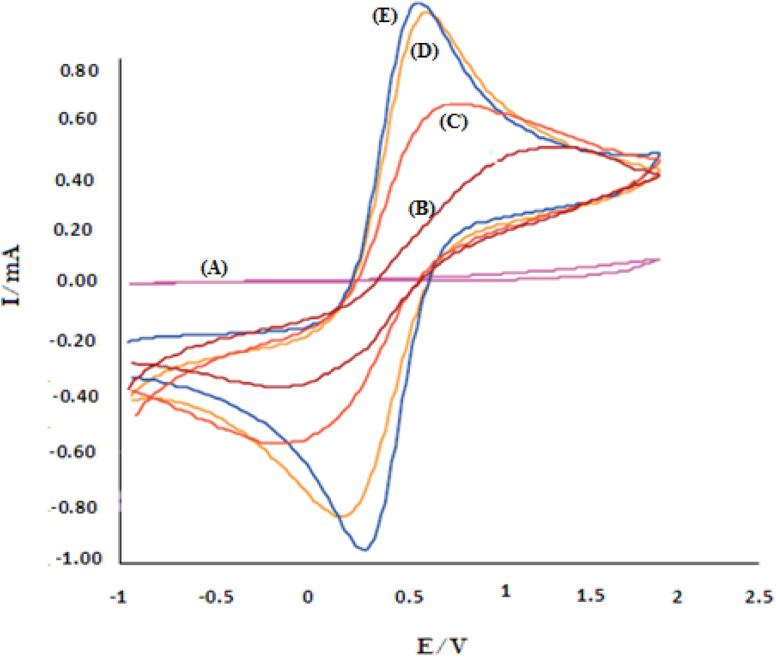
Cyclic voltammograms of (A) Au bare electrode, (B) Au/PPy electrode, (C) Au/PPy + Au NPs electrode, (D) Au/PPy + Au NPs/l-cysteine electrode, and (E) Au/PPy + Au NPs/l-cysteine/ZIF-8 crystalline structure electrode in 20 nM of insulin hormone solution (pH 7) at and scan rate 100 mV s^−1^.

As shown in Fig. 6, the absence of any electrochemical response at the Au bare electrode indicates that the electrooxidation/reduction of 20 nM insulin is difficult to understand in the commonly applied working electrode. Small peaks appeared on the Au/PPy electrode, which could be ascribed to the facile electrochemical oxidation/reduction of insulin. Due to the Au NPs specific properties in the conductive polymer matrix with definite electrocatalytic capability, the electro-reduction of insulin was improved. The reduction peak considerably appeared by visibly enhancing the reduction peak current on Au/Au NPs-PPy/l-CYs/ZIF-8 crystalline structure. The results indicated that the zeolitic imidazolate frameworks (ZIFs) presence in the film sensor exhibited promoting effects for insulin reduction. The appearance of a small reduction peak at +0.20 V (curve B) at the Au/PPy electrode can be attributed to the easy electrochemical reduction of insulin. On Au/PPy + Au NPs/l-cysteine/ZIF-8 crystalline, the reduction peak significantly appeared at +0.40 V with the obvious increase of the reduction peak current (curve E). The results indicated that the presence of ZIF-8 in the film sensor exhibits promoting effects for insulin reduction. The synthetic Au/PPy + Au NPs/l-cysteine/ZIF-8 acts as an electron mediator substance in the film sensor to accelerate the electron transfer of the electrochemical process, and it is worth mentioning that cyclic voltammetry is a reversible process.

### 3.3 Surface characterization of Au/PPy + Au NPs/l-cysteine/ZIF-8 crystalline

**Fig. 7:**
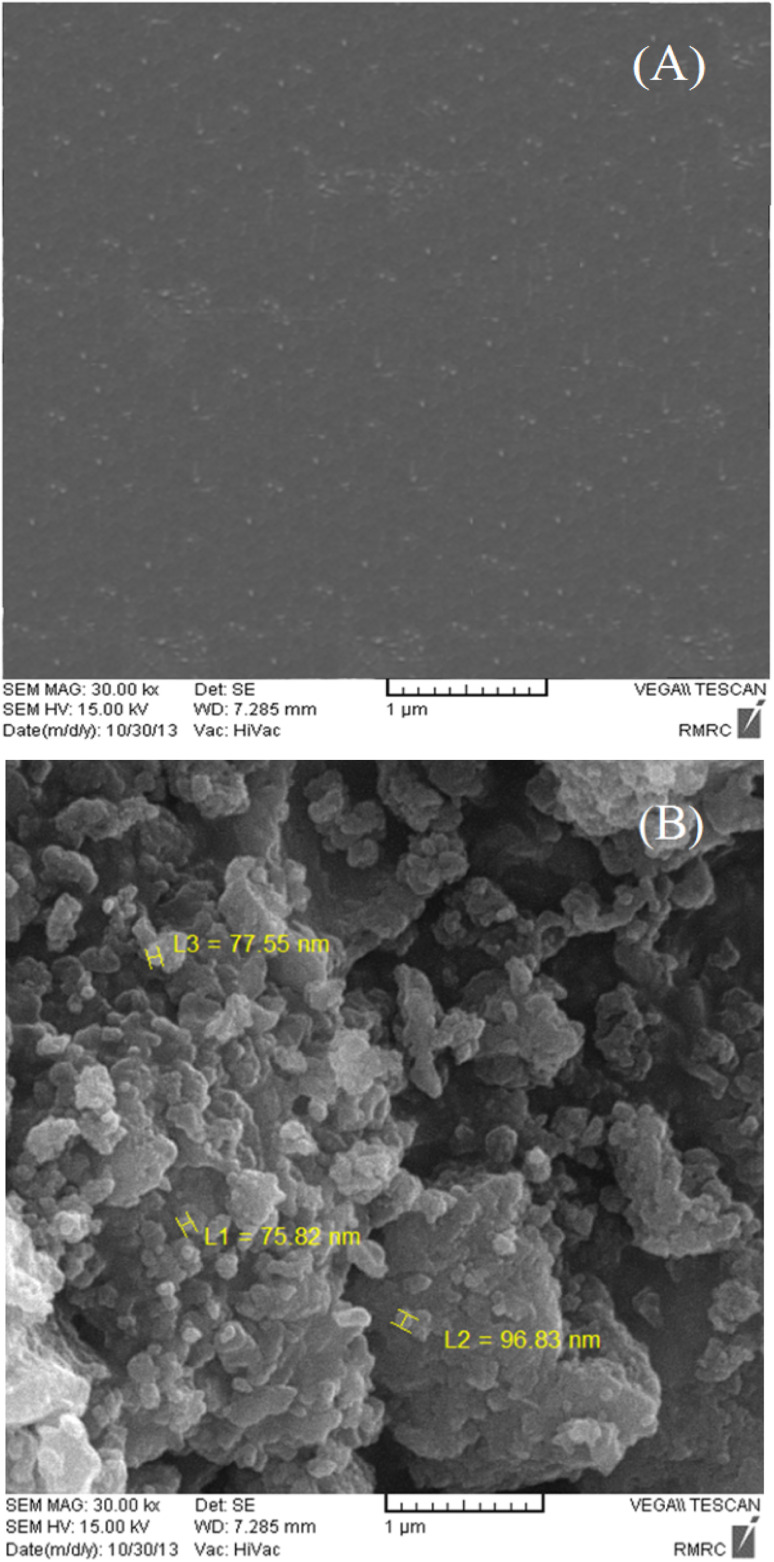
SEM image for bare Au (A) and Au/PPy + Au NPs/l-cysteine/ZIF-8 crystalline (B) modified electrodes.

The morphology of the obtained Au/PPy + Au NPs/l-cysteine/ZIF-8 film was also observed by SEM (Fig. 7). The SEM image of ZIF-8 suggests a smooth morphology and confirms that the particles still fall in the Nano-size range. In addition, the SEM images show the successful polymerization of the matrix on the surface of the gold electrode.

**Fig. 8:**
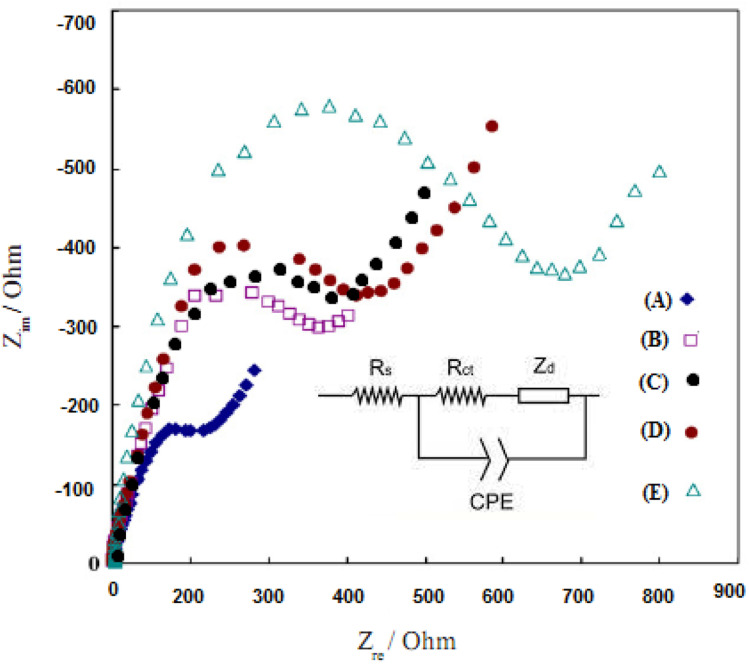
The electrochemical impedance of (A) Au bare electrode, (B) Au/PPy electrode, (C) Au/PPy + Au NPs electrode, (D) Au/PPy + Au NPs/l-cysteine electrode, and (E) Au/PPy + Au NPs/l-cysteine/ZIF-8 crystalline structure in the presence of a solution of 10.00 mmol L^−1^ [Fe(CN)_6_]^3−/4−^, 0.5 mol L^−1^ KI with the frequencies ranging from 100 000 to 0.1 Hz.

Electrochemical Impedance Spectroscopy (EIS) can provide information about the impedance changes of the modified electrodes during the modification process and increase the electrical resistance of the modified layers. Fig. 8 shows the typical EIS results for different modified electrodes in 10.0 mM K_3_Fe(CN)_6_/K_4_Fe(CN)_6_ solution and 0.5 mol L^−1^ KI with frequencies ranging from 100 000 to 0.1 Hz.^53^ The semicircle with a small diameter belongs to the bare electrode of gold, and this small conductivity is due to the inherent properties of gold (Fig. 8, curve A). With the increase of the layers on the surface of the electrode (Fig. 8, B–E curves), the observation of the increase in the diameter of the semicircles in this graph proves the coating layer's success in increasing the efficiency of the electrode. This phenomenon is likely due to the excitation of the PPy and Au/PPy + Au NPs/l-cysteine/ZIF-8 crystalline doped into the film electrodes, which enhances the resistance of the films.

### 3.4 Effect of pH

**Fig. 9:**
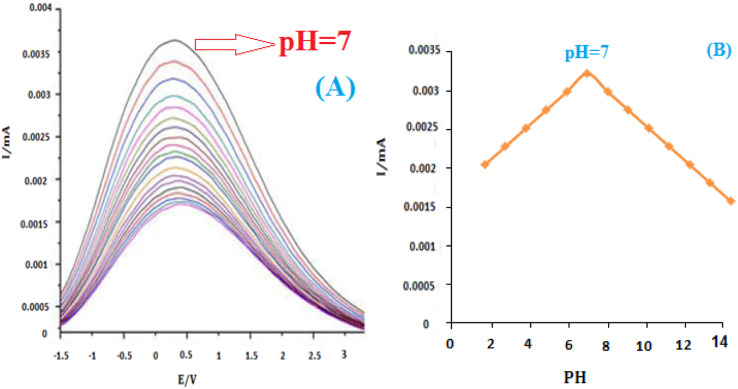
(A) The effect of pH on the reduction peak current response of 20 nM insulin in 2.5 mM 0.1 mol L^−1^ pH 6.0 B–R buffer solution at scan rate 100 mV s^−1^. (B) The plot of E00 *vs.* pH for Au/PPy + Au NPs/l-cysteine/ZIF-8 crystalline modified Au electrode.

The influence of pH on the electrochemical response of insulin hormone was examined with the results shown in (Fig. 9A). A stable and pretty well-defined irreversible oxidation peak was obtained in the pH range of 2.0–12.0. Due to the presence of the C=O group on the structure of insulin hormone, its electrochemical behavior strongly depends on the pH of the test solutions. The oxidation peak currents increased with the increase of buffer pH until it reached 7, then the peak currents decreased gradually with the further increase of pH value. So pH 7.0 B–R buffer was used as the supporting electrolyte in the following experiments.

Fig. 9B shows that the formal value of *E*^0^′ is linear with pHs and a slope of 0.0035 mV pH^−1^ for insulin. These slopes correspond to the electron transfer process for insulin reductions at the surface of the modified electrode. It has been reported that insulin is an essential component of the electron transport chain in mitochondria with the electron transfer process.^54^

### 3.5 Influence of the scan rate

With the increase of the scan rate (100–500 mV s^−1^), the oxidation/reduction peak currents gradually increased with the positive shift of the reduction peak potential, which indicated that the electrode process was irreversible. The oxidation peak current showed a good linear relationship with the scan rate in the range from 100 to 500 mV s^−1^, and the linear regression equation was calculated as *I*_pa_ (μA) = 0.418*θ* (mv s^−1^) + 22.12 (*n* = 2, *δ*= 0.994). The results showed that the electrode process was controlled by adsorption.^55^3
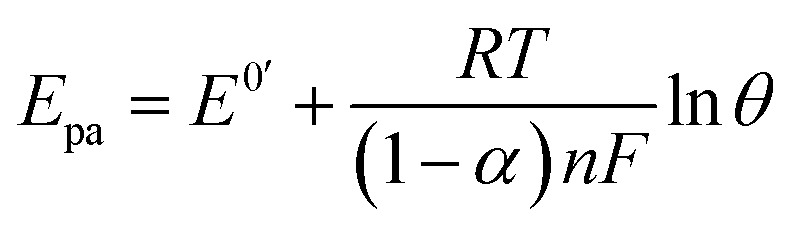
4
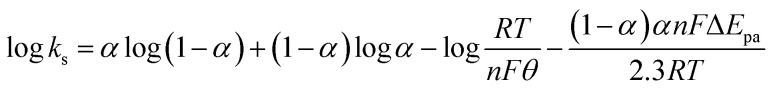


**Fig. 10:**
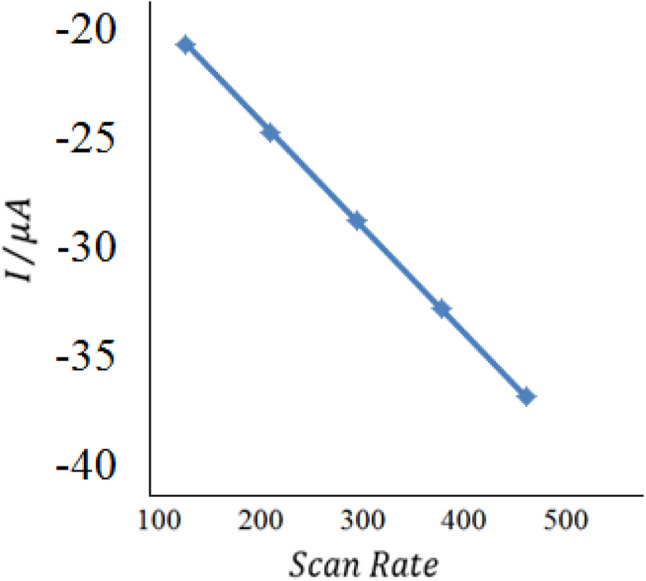
The plot of peak currents *vs.* scan rate (inner to outer) 100, 200, 300, 400, and 500 mV s^−1^ of 20 nM insulin hormone at the modified electrode in pH 7.0 B–R buffer solution.

The electrochemical parameters, such as the charge transfer coefficient (*α*) and the electrode reaction standard rate constant (*k*_s_), were calculated with the results as 0.74 and 3.36 × 10^−4^ s^−1^, respectively. Since the electrode process was controlled by adsorption, the adsorption amount of electroactive insulin hormone on the Au/PPy + Au NPs/l-cysteine/ZIF-8 modified Au electrode was calculated by the down eqn.5
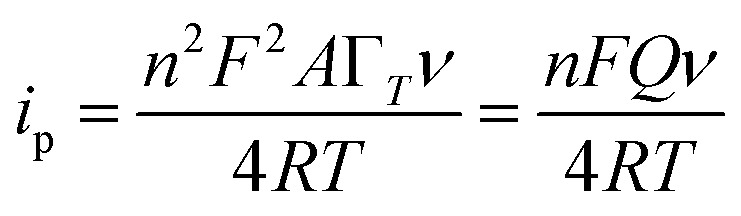
where *n* is the number of electrons transferred, F(C mol^−1^) is Faraday's constant; *A* (cm^2^) is the area of the electrode, *Γ* (mol cm^−2^) is the surface concentration of the electroactive substance, *i.e.*, insulin hormone, *Q* (C) is the peak area (calculated by the charges) and *ν* (V s^−1^) is the scan rate. By integrating the relationship of *i*_p_ with *ν*, the values of *n* and *Γ* were obtained by the results as 1.75 and 7.82 × 10^−10^ mol cm^−2^, respectively. The results indicated that the presence of Au/PPy + Au NPs/l-cysteine/ZIF-8 on the electrode surface provided a nanostructure for the effective electrochemical reduction of insulin hormone on the surface of the final modified electrode, which resulted in the improvement of the sensitivity (Fig. 10).

### 3.6 Calibration curve

**Fig. 11:**
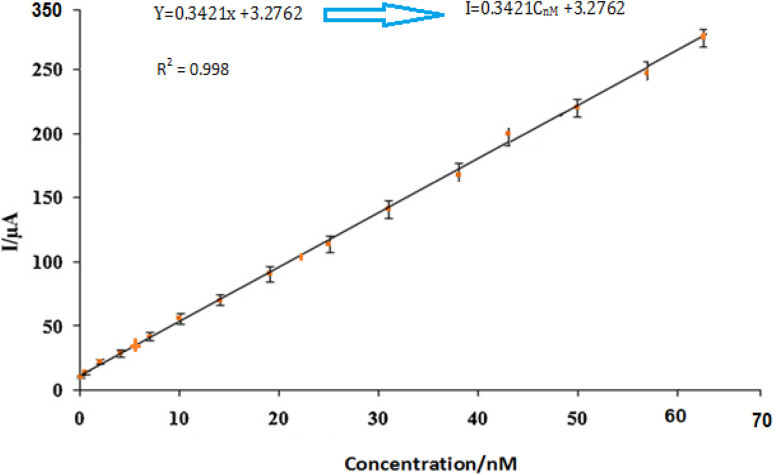
The plot of peak currents *vs.* insulin hormone concentrations.

By using the more sensitive SWV, the proposed electrode was further used for the insulin hormone measurement with the typical voltammograms shown in Fig. 11. Under the optimal conditions, the oxidation peak current of insulin hormone increased with its concentration in the range from 1.0 to 60 nM with the linear regression equation as *I*_pa_ = 0.3421*C* (nM) + 3.2762 (*γ* = 0.998) The measurement limit was estimated to be 1 nM.

### 3.7 Study stability and selectivity sensor

The reproducibility and stability of the sensor were also studied. Seven Au/PPy + Au NPs/l-cysteine/ZIF-8 modified Au electrodes were investigated in 20 nM insulin hormone at a scan rate of 100 mV s^−1^. The relative standard deviation (R.S.D.) was 3.3%, validating that the preparation method was extremely reproducible. The stability of the modified electrode was tested by scanning the electrode continuously in 20 nM insulin hormone. It was shown that there was no significant decrease in the current response for 14 successive consecutive cycles, resulting in good stability of the modified electrode. The long-term stability of the sensor, also assessed after one month by measuring its current response toward the hormone insulin, was 5.6% relative standard deviation (R.S.D.). Selectivity is the most important characteristic of any sensor toward the target ion in the presence of interfering species. This study was conducted under the optimum conditions for 20 nM of insulin hormone. A relative error of 6% concerning the insulin hormone signal was considered tolerable. The results are shown in Table 1.

**Table 1:** This table shows that the proposed sensor is accurate, precise, and free from the side interferences in the applied potential window

Interfering ion	Toleratedratio [interference]/[insulin hormone]	Interfering ion	Tolerated ratio [interference]/[insulin hormone]
Cu^2+^	810	Ni^2+^	741
Zn^2+^	379	Mn^2+^	754
K^+^	914	Co^2+^	854
Vitamin B6	751	Al^3+^	874
Vitamin E	1125	Fe^2+^	752
d-Argenine	795	Glucose, tartarate	981
Sucrose, fructose, glucose	986	CTAB, thiourea, urea	1254

### 3.8 Analytical application of the proposed sensor

To specify the electrochemical response of the proposed sensor towards insulin hormone in the pharmaceutical and human blood serum samples, the samples were pre-treated in the appropriate manners explained and analyzed by the proposed sensor at pH 7. The results are shown in Tables 2 and 3. The results obtained by the proposed sensor agree with the initial values.

**Table 2:** Found value by the proposed sensor (nM)^a^

Sample	Spiked (nM)	Found value by the proposed sensor (nM)	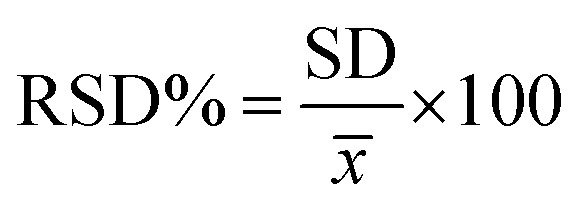
H.B.S.	0	0.98 ± 0.09	9.18
H.B.S.	5	5.53 ± 0.38	6.87
H. B. S.	10	10.70 ± 0.66	6.16
H.B.S.	15	15.90 ± 0.92	5.78
H.B.S.	30	31.54 ± 1.42	4.50
H.B.S.	40	41.90 ± 1.80	4.29

a H.B.S. = human blood serum sample.

**Table 3:** Found Value by the Proposed Sensor (μg mL^−1^)

Sample (vial)	Ingredient	Initial value insulin hormone (μg mL^−1^)	Found value by the proposed sensor (μg mL^−1^)
GentleJintropin insulin hormone (vial) by GeneScience pharmaceuticals Co. Ltd: China	Insulin hormone: 4 i.u./1 vial	1300	1333.7 ± 35.3
Insulin hormone Asia Pharma Co. Ltd: Indonesia (http://www.vitalitypharm.com/asia-pharma.php)	hGH: 4 i.u./1 vial	1300	1328.3 ± 29.7

Different nano-modified carbon electrodes for non-immune insulin detection and their analytical performance, *e.g.*, linear concentration range, limit of detection, pH, and sensitivity, are summarized in Table 4.

**Table 4:** Nano-modified electrodes for non-immune insulin detection and their analytical performances^a^

Electrode matrix	Linear concentration range	Limit of detection	Sensitivity	Size of nanoparticles	pH	Reference
SiO_2_NPs/CE	10–560 nM	36 pM	107.3 pA pM^−1^	—	7.4	56
rGO/GCE	4–640 nM	350 pM	7.1254 nA nM^−1^	—	7.4	57
Ni(OH)_2_-GN/GCE	800–6400 nM	200 nM	—	—	11	58
NiONPs-Guanin/GCE	4 μM	22 pM	100.9 pA pM^−1^	70–250 nm	7.4	59
SiCNPs-Nafion/GCE	600 pM	3.3 pM	710 pA pM cm^−2^	<20 nm	7.4	60
SiO_2_NPs-Nafion/GCE	—	3.1 nM	300 pA nM^−1^	30–40 nm	7.3	61
CNTs-Chitosan/GCE	0.1–3 μM	30 nM	135 mA M cm^−2^	—	7.4	62
RuO-CNTs/GCE	10–800 nM	1 nM	—	40-nm diameter	7.4	63
MWCNTs-NiCoO_2_-Nafion/SPCE	0.1–31.5 μg mL^−1^	0.22 μg mL^−1^	22.57 μA mg^−1^ mL^−1^	6–13 diameter	7.5	64
NiONPs-Nafion-MWCNTs/SPCE	20–260 nM	6.1 nM	1.83 mA mM^−1^	<30 nm (NiONPs)	7.4	65
Au/PPy + Au NPs/l-cysteine/ZIF-8 crystalline	1.0–60 nM	1 nM	7.82 × 10^−10^ mol cm^−2^	<100 nm	7.0	This work

a AgNF silver nanoflower, CE carbon electrode, CNTs carbon nanotubes, GN graphene, ITO indium tin oxide, rGO reduced graphene oxide, SPCE screen printed carbon electrode.

## 4 Conclusions

Zeolitic imidazolate framework-8 (ZIF-8) was successfully synthesized and identified in this study. Next, the fabricated electrochemical nanosensor was tested by reducing the oxidation potential and improving the current conductivity to increase the selectivity and sensitivity in identifying insulin hormones in human biological samples. The results were found to be successful. Also, side interferences and stability of the electrode were investigated, which shows the suitability of this system and its applicability in similar projects. This study was conducted under the optimum conditions for 20 nM of insulin hormone. A relative error of 6% concerning the insulin hormone signal was considered tolerable.

## Author contributions

Prof. Hassan Keypour: project supervisor Dr Khadijeh Rabiei: synthesis and identification of materials and participation in the project. Jamal Kouhdareh: PhD student inorganic chemistry, synthesis and identification of materials and participation in the project. Electrochemical analysis Dr Sedigheh Alavinia: synthesis and identification of materials and participation in the project. Dr Rahman Karimi-Nami: consultant in project and data analysis. Dr Shokoufeh Ghahri Saremi: consultant in project and data analysis. Dr Mohammad Noroozi: consultant in project and data analysis.

## Conflicts of interest

The authors declare that they have no competing interests.

## Supplementary Material
